# Synthesis of Iron Nanoparticles from *Spartina alterniflora* for Cadmium Immobilization in Coastal Wetland Sediments

**DOI:** 10.3390/biology14111626

**Published:** 2025-11-19

**Authors:** Jian Li, Xuejing Zang, Zhanrui Leng, Yan Li, Shiyan Xu, Na Wei

**Affiliations:** Institute of Environment and Ecology, School of the Environment and Safety Engineering, Jiangsu University, Zhenjiang 212013, China; 2222209077@stmail.ujs.edu.cn (X.Z.); zhanruileng@gmail.com (Z.L.); liyanhellen@ujs.edu.cn (Y.L.); 2222309006@stmail.ujs.edu.cn (S.X.); 2222309008@stmail.ujs.edu.cn (N.W.)

**Keywords:** *Spartina alterniflora*, iron nanoparticles, cadmium, coastal wetlands

## Abstract

A green synthesis method for iron nanoparticles was developed using the leaf extract of the invasive plant *Spartina alterniflora* (Sa-FeNPs), targeting the remediation of cadmium (Cd) in coastal sediments. The obtained Sa-FeNPs, characterized as amorphous Fe-O-C composites, significantly reduced the amount of bioavailable Cd. Specifically, Sa-FeNPs achieved a 34.63–38.39% decrease in the weak-acid-extractable fraction at a 7% application rate. The primary mechanisms underlying the adsorption and complexation of Cd are the large specific surface area of the Sa-FeNPs, their rich functional groups (-OH, -COOH, C=C, C-O), iron redox reactions, and total organic carbon (TOC) conversion. This study offers an environmentally friendly and low-cost remediation strategy for the utilization of excessive *S. alterniflora* biomass resources and controlling Cd pollution in coastal wetlands.

## Simple Summary

In this research, we tackled two environmental issues in coastal wetlands-cadmium (Cd) contamination and excessive *S. alterniflora* biomass-with a sustainable strategy. We converted this invasive plant into iron-based nanoparticles (Sa-FeNPs) to immobilize the toxic metal. Experiments demonstrated that Sa-FeNPs significantly reduced the hazardous bioavailable fraction of Cd in sediments. The nanoparticles act by trapping Cd and promoting its conversion into a stable, immobile state. Our work presents a circular economy solution, turning an ecological problem into a tool for decontamination, thereby promoting sustainable coastal management.

## 1. Introduction

The perennial herb *Spartina alterniflora* was introduced to China in 1979 for storm surge protection, land reclamation, and coastal erosion control. However, its robust survival characteristics-such as a well-developed root system, salt tolerance, flood resistance, strong adaptability, prolific reproductive capacity, and invasive potential-have enabled rapid expansion across China’s coastal regions. It is now recognized as one of the most invasive species in China’s coastal wetlands, threatening local ecosystems and biodiversity. This expansion has led to significant environmental degradation and economic losses. Large-scale removal of *S. alterniflora* generates substantial biomass waste. If this waste is directly landfilled or incinerated, it not only leads to resource waste but also may cause secondary environmental issues. However, *S. alterniflora* is a versatile plant resource: its extracts exhibit allelopathic effects that inhibit harmful alga growth in aquatic environments [1]. It can also serve as feed for ruminants and fulfill other utilitarian purposes [2]. Furthermore, *S. alterniflora* can be converted into biochar for remediating heavy metal pollution and improving soil quality [3]. As a result, the exploitation of *S. alterniflora* resources is emerging as a key focus for its future management.

Iron nanoparticles, with their large specific surface area and high reactivity, show significant potential for adsorbing organic and inorganic pollutants, leading to their widespread applications in soil and water pollution remediation. However, traditional iron nanoparticle synthesis methods often rely on borohydride reduction, which involves toxic chemicals and solvents, incurs substantial costs, and results in harmful residues [4]. Furthermore, iron nanoparticles are prone to oxidation and agglomeration, which reduces their specific surface area and activity. An innovative green synthesis method using plant extracts offers a solution to these challenges, eliminating the need for pressure, high temperatures, and toxic chemicals [5]. Plant extracts contain phenolic compounds, flavonoids, alkaloids, reducing sugars, and other bioactive substances that act as reducing agents to convert iron ions (from dissolved iron salts) into zero-valent iron during nanoparticle synthesis through chemical reduction. The reduced iron atoms coalesce to form stable nuclei. The continued supply of reducing agents from the extract promotes the growth of these nuclei into nanocrystals. Subsequently, excess biomolecules such as proteins spontaneously encapsulate the nanoparticles, stabilizing them through a capping effect [6]. As a result, the green-synthesized iron nanoparticles exhibit reduced toxicity and agglomeration, show enhanced stability, and are environmentally friendly. The chemical synthesis method mediated by plant extracts not only minimizes the risks to human health and ecosystems to the greatest extent, but is also cost-effective.

Due to the advantages of a simplified process, ease of operation, low energy consumption, and environmental friendliness, the green synthesis of iron nanoparticles has become a focal point in environmental pollution remediation research. Extracts from various plant parts-including leaves, stems, roots, fruits, peels, seeds, and other plant waste-have been successfully used to synthesize iron nanoparticles. Specifically, green iron nanoparticles synthesized from green tea and eucalyptus leaf extracts have proven effective in removing nitrate, Cr (VI), dyes, and other pollutants from aqueous solutions [7]. Twenty-six types of leaf extracts were used to prepare green zero-valent iron nanoparticles, and it was found that those derived from wolfberry and vine leaves exhibited superior performance in remediating Cr (VI) pollution in water [8]. Additionally, nano-sized zero-valent iron particles (nZVIs) synthesized from black tea, grape residue, and vine leaves were successfully used for remediating ibuprofen-contaminated soil [9]. Beyond the selection of synthetic materials, the application of green iron nanoparticles (FeNPs) requires particular attention. At present, the application of green FeNPs is primarily focused on water pollutant treatment. Compared with water bodies, soils and sediments not only have higher organic matter contents and more complex pore structures but also contain various colloids and mineral particles, all of which can affect the aggregation and migration of FeNPs and their interactions with heavy metals. Prior studies mostly focused on agricultural soils; however, the systematic exploration of FeNP application in typically contaminated sediments in high-salinity, low-oxygen environments (such as estuaries, salt marshes, and wetlands) remains lacking. Coastal wetlands, serving as critical transition zones between terrestrial and marine ecosystems, have become significant sinks for heavy metal pollutants [10,11]. Plant-synthesized iron nanoparticles have demonstrated high removal capacities for cadmium (Cd) [12], arsenic (As) [13], and chromium (Cr) [7,8] from contaminated water and soil. These studies collectively underscore the broad potential of green nanotechnology for multi-metal remediation. However, among these pollutants, cadmium (Cd) is a persistent, bioaccumulative toxic metal whose severe toxicity necessitates urgent remediation [14]. Its chronic toxicity severely impacts human health by causing kidney and skeletal damage and being carcinogenic [15]. Concurrently, Cd impairs ecosystems by disrupting microbial and plant life and causing oxidative stress in organisms [16]. The persistence and bioaccumulative nature of Cd make its immobilization in sediments a critical priority for environmental and public health protection [10].

*S. alterniflora*, an invasive plant with high biomass in coastal wetlands, is rich in phenolic and flavonoid compounds, exhibiting potent antioxidant activity [17]. This makes it a sustainable source of bioactive substances and a promising candidate for synthesizing green iron nanoparticles. However, there are no reports to date on the preparation of green iron nanoparticles from *S. alterniflora*. This study aims to (1) chemically synthesize green FeNPs using *S. alterniflora* extract; (2) evaluate the efficacy of these FeNPs in remediating Cd pollution in coastal wetlands; and (3) investigate the underlying remediation mechanisms.

## 2. Materials and Methods

### 2.1. Sediments and Materials

*S. alterniflora* leaves and surface sediments (20–30 cm depth) were collected from the Liangduo River estuary (32°52′ N, 120°54′ E), Dongtai City, Jiangsu Province, China. Sediment properties at a depth of 20–30 cm are more uniform and stable, are less affected by short-term environmental fluctuations, and better represent the intrinsic properties of sediments. After all animal and plant residues were removed, distilled water was added to the sediment samples to ensure thorough mixing and homogenization in a moist state. The measured pH of the sediment was 4.82 ± 0.07, the electrical conductivity (EC) was 404.67 ± 9.07 μS/cm, the organic matter (OM) content was 8.90 ± 0.05 g/kg, and the total Cd concentration was 0.76 ± 0.01 mg kg^−1^.

### 2.2. Synthesis and Characterization of Sa-FeNPs

After harvesting, the leaves of *S. alterniflora* were cleansed with deionized water to remove surface contaminants. The samples were then pre-cooled in liquid nitrogen and subsequently freeze-dried at −60 °C using a freeze dryer (LC-10N-50A, LICHEN, Shanghai, China). The dried leaves were ground into a fine powder using a stone tea mill to prevent thermal degradation of the active compounds, and the powder was sieved through a 60-mesh screen. A 30 g aliquot of the powder was subjected to ultrasonic extraction with 500 mL of ultrapure water at 30 °C for 50 min. After cooling to room temperature, the mixture was vacuum-filtered through a 0.45 μm filter membrane to collect the filtrate. Under an Ar atmosphere and continuous stirring, 250 mL of FeCl_3_·6H_2_O (0.1 M) was slowly added dropwise to 500 mL of the *S. alterniflora* leaf extract in a three-necked flask. Stirring was continued at room temperature for an additional 30 min after the addition was complete. The resulting black precipitate was collected by means of filtration and washed three times each with deionized water and absolute ethanol. Finally, the precipitate was freeze-dried to obtain iron nanoparticles synthesized from *S. alterniflora* leaf extract (Sa-FeNPs).

The morphology, size, and composition of the Sa-FeNPs were characterized using field-emission scanning electron microscopy (SEM, JSM-7800F, JEOL Ltd., Tokyo, Japan) coupled with energy-dispersive X-ray spectrometry (EDS). The internal microstructure was further examined using high-resolution transmission electron microscopy (HR-TEM, JEM-2100, JEOL Ltd, Tokyo, Japan). The crystalline phase composition of the Sa-FeNPs was analyzed by means of X-ray diffraction (XRD) using a multifunctional X-ray diffractometer (SmartLab, Rigaku Corporation, Tokyo, Japan), with scans conducted at a rate of 8° min^−1^ over a 2θ range of 10–90°. The surface chemical states and atomic composition were investigated using X-ray photoelectron spectroscopy (XPS) on a multifunctional X-ray photoelectron spectrometer (ESCALAB QXi, Thermo Fisher Scientific, Waltham, MA, USA). The functional groups present on the surface of the Sa-FeNPs were identified via Fourier transform infrared (FTIR) spectroscopy. The specific surface area of the Sa-FeNPs was determined using a specific surface area and porosity analyzer (TriStar II 3020, Micromeritics Instrument Corporation, Norcross, GA, USA).

### 2.3. Experimental Design

A sediment incubation experiment was established as a fully crossed design with three levels of Cd (0, 2, 8 mg kg^−1^ DW) and three levels of Sa-FeNPs (0, 1, 7% *w*/*w*). The 0% Sa-FeNP treatment for each Cd level constituted the control. Each of the 9 treatment combinations was replicated three times, resulting in 27 independent samples (*n* = 27). For each sample, 40 g (DW) of sediment was maintained at a 50% water content using ultrapure water in a 100 mL beaker. The beakers were sealed with Parafilm and aluminum foil and incubated at 25 °C for one month. Cd concentrations were determined based on our previous research on heavy metal pollution in coastal wetlands [11]. The Sa-FeNP doses were selected based on prior research [18] and preliminary tests confirming Cd immobilization efficacy. Following the culture process, the sediment was collected and subsequently freeze-dried. The dried samples were ground and sieved (<2 mm) prior to analysis.

### 2.4. Chemical Analysis

Weak-acid-extractable Cd, reducible Cd, oxidizable Cd, and residual Cd were fractionated using the BCR sequential extraction procedure for heavy metals. The sequential extraction was performed as follows: Step 1: Extraction with acetic acid (0.11 mol/L) to isolate the acid-soluble fraction (exchangeable and carbonate-bound Cd). Step 2: Extraction with hydroxylamine hydrochloride (0.5 mol/L, pH 2) to obtain the reducible fraction (Cd bound to Fe/Mn oxides). Step 3: Digestion with hydrogen peroxide followed by extraction with ammonium acetate to separate the oxidizable fraction (Cd associated with organic matter and sulfides). Residual Cd was determined by subtracting the sum of the three BCR fractions from the total Cd content [19]. The total Cd content was calculated according to the method described by Li and Liu [20]. The supernatants obtained from each extraction step were filtered through a 0.45 μm membrane filter, and the Cd concentration was determined using flame atomic absorption spectrometry (AA-6800, Shimadzu, Kyoto, Japan). Free iron oxides (Fed) were extracted using a sodium dithionite-citrate-bicarbonate solution. Amorphous iron oxides (Feo) were isolated with 0.2 M ammonium oxalate (pH 3.0), while complex iron oxides (Fep) were extracted with 0.1 M sodium pyrophosphate (pH 8.5). Fe(II) was quantified after extraction with 0.5 M HCl for 24 h. Fe(III) was measured after reduction to Fe(II) using 10% hydroxylamine hydrochloride [21]. The extracted Fe content was then measured at 510 nm using the 1,10-phenanthroline method on a spectrophotometer (UV-1800PC, Mapada, Shanghai, China) [22]. Total organic carbon (TOC) was determined via potassium dichromate oxidation spectrophotometry [23]. The content of soil organic matter (OM) was calculated from the measured TOC value using the conventional conversion coefficient in soil science (OM = TOC × 1.724) [24]. Dissolved organic carbon (DOC) was extracted by shaking a 5:1 suspension of Milli-Q water and soil at 250 rpm for 60 min, followed by centrifugation (3000× *g*, 20 min) and filtration. DOC concentrations were analyzed using a total organic carbon analyzer (TOC-L, Shimadzu) [12]. Iron-bound organic carbon (Fe-OC) in the sediments was determined using the sodium disulfite-citrate–bicarbonate reduction method. The sediment pH was measured using a pH meter (PSHJ-4F, INESA, Shanghai, China) [25].

### 2.5. Data Analysis

Statistical analysis was conducted using IBM SPSS Statistics 27.0 (IBM SPSS Statistics for Windows, IBM Corporation, Armonk, NY, USA). Following Levene’s test for homogeneity of variances, one-way ANOVA was employed to assess the differences in soil properties and Cd fractions across various treatments. Significant differences were indicated by different letters when *p* < 0.05. Pearson correlation analysis was utilized to investigate the relationships between each treatment and environmental factors. The diagrams were generated using Origin 2024b (OriginLab, Northampton, MA, USA).

## 3. Results

### 3.1. Characterization of Sa-FeNPs

**Figure 1:**
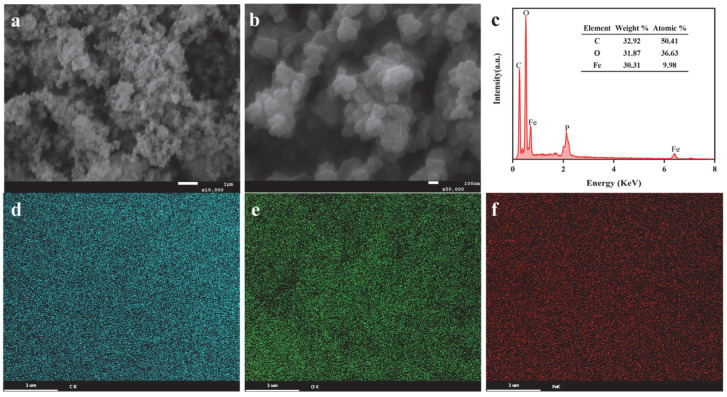
(**a**,**b**) SEM images of Sa-FeNPs, (**c**) their EDS spectrum, and the corresponding elemental maps of (**d**) C, (**e**) O, and (**f**) Fe.

The morphology, size, and elemental composition of the Sa-FeNPs were characterized using SEM-EDS. As shown in Figure 1, the SEM images (a,b) and EDS spectrum (c) confirmed the successful synthesis of the nanoparticles. The newly synthesized Sa-FeNPs exhibited irregular spherical shapes and were moderately aggregated into clusters, with particle sizes ranging from 34 to 119 nm and an average particle size of 67.9 ± 17.8 nm. The observed heterogeneity in the Sa-FeNPs can be attributed to the plant extract being a mixture of organic compounds with varying reducing properties. BET measurements revealed a specific surface area of 38.7116 m^2^/g, which is slightly higher than that of iron nanoparticles synthesized from green tea extract (37.08 m^2^/g) [13] and significantly higher than that of iron-based nanoparticles prepared from yerba mate extract (17.52 m^2^/g) [26].

To gain a deeper understanding of the synthesis and composition of the Sa-FeNPs, their surface elemental distribution was characterized using EDS, as illustrated in Figure 1c–f. In Figure 1c, prominent peaks for C, O, and Fe can be observed, confirming the successful formation of iron nanoparticles. The elemental mapping images (Figure 1d–f) revealed that C, O, and Fe were closely integrated, forming an Fe-O-C macromolecular complex. Specifically, the atomic percentages of C, O, and Fe in the Sa-FeNPs were 32.92%, 31.87%, and 30.31%, respectively (Figure 1c). The presence of C and O primarily originates from phenolic, flavonoid, and sugar molecules in the *S. alterniflora* leaf extract [17]. However, given that Fe^0^ is highly reactive with air and water, leading to the formation of an iron oxide shell, the O signal may also indicate the presence of some iron oxide nanoparticles. Previous studies have reported Fe loadings of 27.8% and 16.17% for iron nanoparticles synthesized from green tea and eucalyptus leaf extracts, respectively [27], which are lower than that observed in Sa-FeNPs.

**Figure 2:**
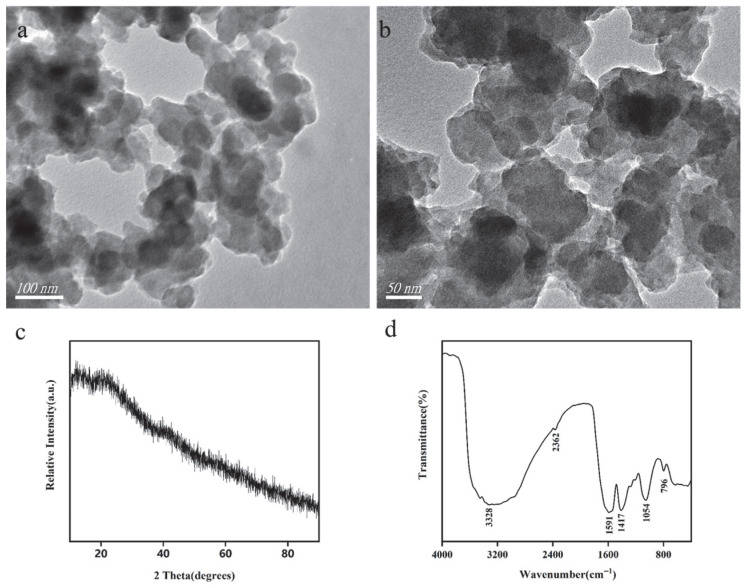
(**a**,**b**) TEM, (**c**) XRD, and (**d**) FTIR images of Sa-FeNPs.

To gain a comprehensive understanding of the detailed morphology of the Sa-FeNPs, transmission electron microscopy (TEM) analysis was performed. The TEM images revealed that the Sa-FeNPs formed irregular chain-like aggregates with sizes within 100 nm, resulting in dense clusters of iron/organic composite materials (Figure 2a,b). Notably, black particles observed within these aggregates were identified as Fe^0^, indicating the successful reduction of iron salts to metallic iron by the active compounds in the *S. alterniflora* extract. This encapsulation process endows the Sa-FeNPs with a distinct core–shell structure.

The XRD pattern of the Sa-FeNPs is presented in Figure 2c. The diffraction pattern lacks distinct and sharp peaks, instead showing a broad hump, which indicates that Sa-FeNPs are predominantly amorphous. A faint characteristic peak corresponding to zero-valent iron (α-Fe) was observed at approximately 2θ = 44.8°. The broad shoulder peak at 2θ = 20° can be attributed to the presence of organic compounds from the leaf extract. These findings confirm that the surface of Sa-FeNPs is coated with organic substances. This is consistent with reports on similar materials: iron nanoparticles synthesized from Fe(II) or Fe(III) salts at room temperature and under acidic pH conditions typically exhibit an amorphous structure, without discernible peaks for Fe^0^ or iron oxides in the XRD pattern and no ferromagnetic behavior [26].

The functional groups on the surface of the Sa-FeNPs were further characterized by recording the transmission spectra from 400 to 4000 cm^−1^ using Fourier transform infrared (FTIR) spectroscopy (Figure 2d). The FTIR spectrum revealed a broad band in the range of 3000–3800 cm^−1^, centered at 3328 cm^−1^, which corresponds to the O-H stretching vibrations of hydroxyl (-OH) or carboxyl (-COOH) groups. Characteristic peaks at approximately 1591 cm^−1^ and 1417 cm^−1^ are attributed to the C=C stretching vibrations of aromatic rings in phenolic compounds such as flavonoids and polyphenols [28]. The peak at around 1054 cm^−1^ is indicative of C-O stretching vibrations, while the peak at approximately 796 cm^−1^ corresponds to the Fe-O stretching vibration [26]. Collectively, these FTIR results confirm the presence of organic functional groups on the surface of Sa-FeNPs, providing evidence that bioactive substances from the extract coat the nanoparticles.

**Figure 3:**
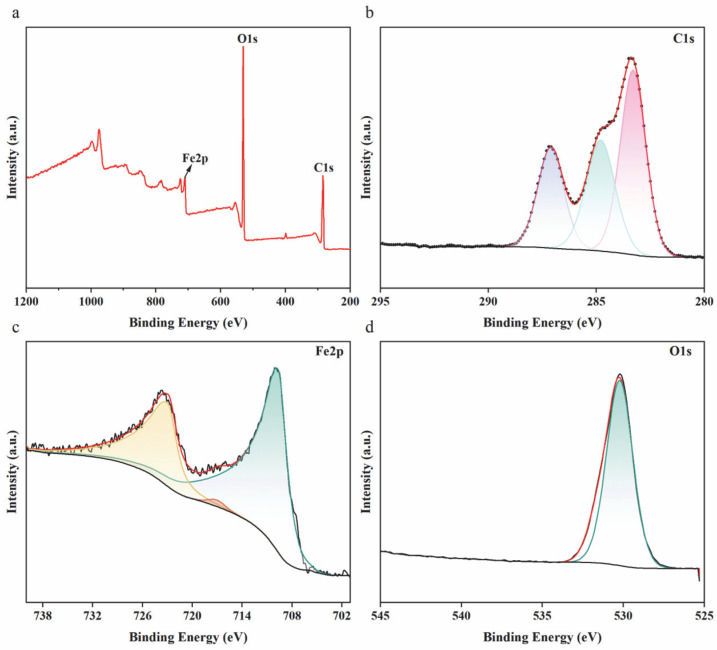
XPS spectra of Sa-FeNPs: (**a**) full XPS survey, (**b**) C1s, (**c**) O1s, and (**d**) Fe2p.

The C1s peak was calibrated to 284.8 eV as an internal standard, yielding the complete XPS survey spectrum and high-resolution spectra of C1s (Figure 3b), O1s (Figure 3c), and Fe 2p (Figure 3d). These spectra reveal the chemical composition and oxidation states of elements on the surface of the Sa-FeNPs. The C1s XPS spectrum (Figure 3b) can be deconvoluted into three characteristic peaks: iron–carbon alloy at 283.3 eV [29], C-C (284.8 eV) [7], and C=C (287.11 eV) [30]. The O1s XPS spectrum (Figure 3c) exhibits a single peak corresponding to O=C-O at 531.69 eV. The Fe2p spectrum (Figure 3d) shows two main peaks at 709.68 eV and 722.94 eV, attributed to FeO and FeOOH [29], respectively, with an additional Fe^3+^ peak at 716.99 eV due to bioactive substance complexation with iron [13]. The C1s, O1s, and Fe2p spectra collectively indicate that the surface of Sa-FeNPs is complex, covered by an oxide film, iron ions, and various carbon coordination compounds.

### 3.2. Effect of Sa-FeNPs on Cd Fractions

**Figure 4:**
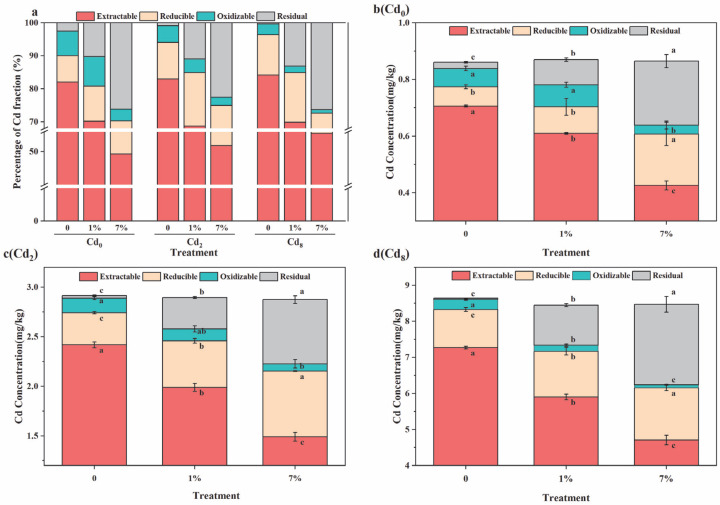
(**a**) Percentages of different Cd fractions in sediments subjected to various treatments; the concentrations of each Cd fraction in sediments treated with different amounts of Sa-FeNPs for initial Cd concentrations of (**b**) Cd_0_, (**c**) Cd_2_, and (**d**) Cd_8_. Cd_0_, Cd_2_, and Cd_8_ represent Cd treatments at 0, 2, and 8 mg kg^−1^ (DW), respectively, while 0, 1%, and 7% represent Sa-FeNP treatments at doses of 0%, 1%, and 7% (wt%), respectively (indicating the Cd or Sa-FeNP content in the sediment). Different lowercase letters indicate significant differences between different Sa-FeNP treatments under the same Cd treatment as determined by the LSD test (*p* < 0.05, *n* = 3), while the same lowercase letters indicate no significant difference.

Figure 4 illustrates the distributions of Cd fractions in sediments with three different Cd levels (0, 2, and 8 mg kg^−1^) after one month of treatment with varying doses of Sa-FeNPs (0%, 1%, and 7%). In sediments of every Cd level, the concentrations of weak-acid-extractable Cd and oxidizable Cd significantly decreased as the Sa-FeNP dose increased, while the concentrations of reducible Cd and residual Cd significantly increased. These changes in Cd fractions indicate that the Sa-FeNPs effectively transformed Cd from highly bioavailable forms to less bioavailable residual forms, thereby reducing its environmental risk. Compared to the control group, both the 1% and 7% Sa-FeNP treatments significantly promoted the transformation of Cd from high-bioavailability forms (weak-acid-extractable) to low-bioavailability forms (reducible, oxidizable, and residual). For Cd_2_ sediments, the concentration of weak-acid-extractable Cd decreased from 2.42 ± 0.003 mg kg^−1^ to 1.99 ± 0.04 mg kg^−1^ and 1.49 ± 0.04 mg kg^−1^ under 1% and 7% Sa-FeNP treatments, respectively, representing reductions of 11.91% and 32.75%, along with significant decreases of 17.74% and 38.39% (*p* < 0.05, *n* = 3). For Cd_8_ sediments, the concentration of weak-acid-extractable Cd decreased from 7.27 ± 0.04 mg kg^−1^ to 5.90 ± 0.07 mg kg^−1^ and 4.71 ± 0.13 mg kg^−1^ under the 1% and 7% Sa-FeNP treatments, respectively, representing reductions of 14.31% and 28.62%, along with significant decreases of 18.86% and 35.3% (*p* < 0.05, *n* = 3). Under both the 1% and 7% Sa-FeNP treatments, the concentration of reducible Cd in sediments of every Cd level increased significantly. In Cd_0_ sediments, the concentration of reducible Cd increased by 2.04 times and 2.73 times, respectively. In Cd_2_ sediments, the concentration of reducible Cd increased by 1.55 times and 2.7 times, respectively. In Cd_8_ sediments, the concentration of reducible Cd increased by 1.23 times and 1.4 times, respectively. For oxidizable Cd, no significant changes were observed under the 1% Sa-FeNP treatment, but significant reductions of 52.09%, 50.06%, and 68.98% were observed under the 7% Sa-FeNP treatment for the Cd_0_, Cd_2_, and Cd_8_ sediments, respectively. The concentration of residual Cd increased with an increase in the Sa-FeNP dose. Under the 1% Sa-FeNP treatment, the residual Cd concentration increased significantly by 7.72%, 10.08%, and 12.77% for the Cd_0_, Cd_2_, and Cd_8_ sediments, respectively. Under the 7% Sa-FeNP treatment, the residual Cd concentration increased significantly by 23.68%, 21.66%, and 25.98% for the Cd_0_, Cd_2_, and Cd_8_ sediments, respectively.

### 3.3. Effect of Sa-FeNPs on Fe Transformation

**Figure 5:**
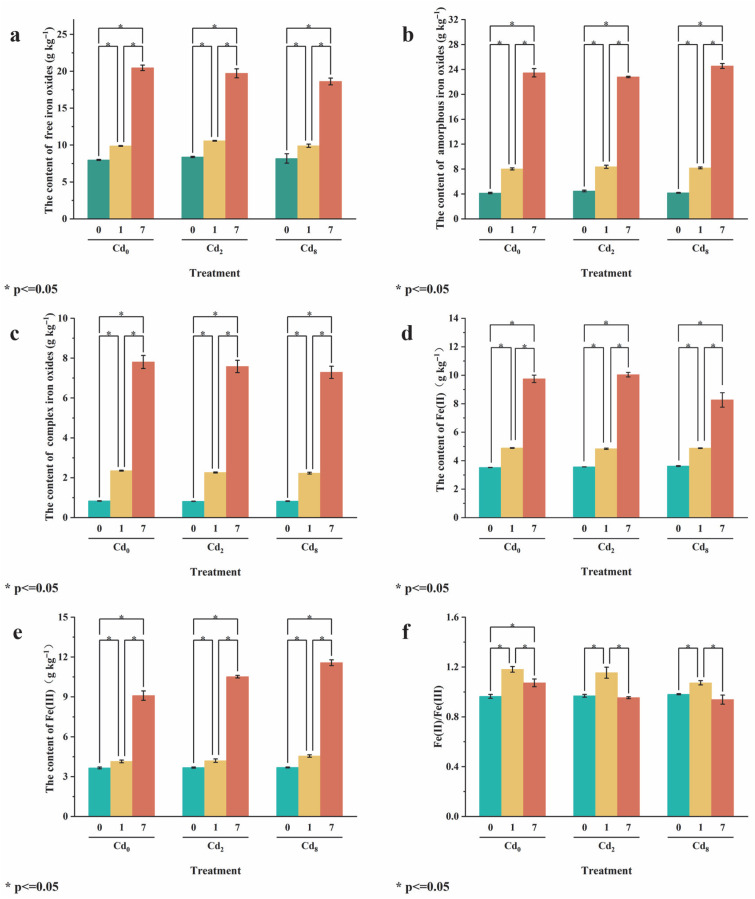
(**a**) The contents of free iron oxides (Fed), (**b**) amorphous iron oxides (Feo), (**c**) complex iron oxides (Fep), (**d**) Fe(II), (**e**) Fe(III), and (**f**) Fe(II)/Fe(III) in the sediments under different treatments. Here, 0, 1, and 7 represent Sa-FeNP treatments at doses of 0%, 1%, and 7% (wt%), respectively (indicating the Sa-FeNP content in the sediment). The symbol * indicates a significant difference in Fe concentration between different Sa-FeNP treatments under the same Cd treatment, as determined by the LSD test (*p* < 0.05, *n* = 3).

The transformation of Fe under different treatments is shown in Figure 5. In sediments with 0, 2, or 8 mg kg^−1^ Cd and without added Sa-FeNPs, the contents of free iron oxide (Fed) were the highest, being 7.94 mg kg^−1^, 8.39 mg kg^−1^, and 8.19 mg kg^−1^, respectively. The contents of amorphous iron oxide (Feo) were the second highest, being 4.15 mg kg^−1^, 4.48 mg kg^−1^, and 4.18 mg kg^−1^, respectively. The contents of complexed iron oxide (Fep) were the lowest. The contents of all iron oxides in sediments of every Cd level significantly increased with an increase in Sa-FeNP addition, especially those of Fed and Feo. Under the treatment with 1% Sa-FeNPs, they increased by 1.95 and 3.87 mg kg^−1^ (Cd_0_), 2.19 and 3.88 mg kg^−1^ (Cd_2_), and 1.72 and 4.01 mg kg^−1^ (Cd_8_), respectively, reaching levels 1.25 and 1.93 times (Cd_0_), 1.26 and 1.87 times (Cd_2_), and 1.21 and 1.96 times (Cd_8_) those of the control group (0% Sa-FeNPs) (*p* < 0.05, *n* = 3). Under the treatment with 7% Sa-FeNPs, Fed and Feo contents were 2.58 and 5.65 times (Cd_0_), 2.35 and 5.09 times (Cd_2_), and 2.27 and 5.87 times (Cd_8_) those of the control group (0% Sa-FeNPs) (*p* < 0.05, *n* = 3).

The contents of Fe(II) and Fe(III) in sediments of every Cd level (Cd_0_, Cd_2_, and Cd_8_) also significantly increased with the addition of Sa-FeNPs (Figure 5d,e). Under the treatment with 1% Sa-FeNPs, the Fe(II) content increased by 1.37 mg kg^−1^, 1.27 mg kg^−1^, and 1.26 mg kg^−1^ while the Fe(III) content increased by 0.49 mg kg^−1^, 0.52 mg kg^−1^, and 0.86 mg kg^−1^ for Cd_0_, Cd_2_, and Cd_8_ sediments, respectively. The corresponding Fe(II)/Fe(III) ratios also significantly increased (*p* < 0.05, *n* = 3). Under the treatment with 7% Sa-FeNPs, the Fe(II) and Fe(III) contents of sediments of every Cd level increased by 5.98–6.48 mg kg^−1^ and 5.44–6.84 mg kg^−1^, respectively. However, the corresponding Fe(II)/Fe(III) ratios did not change significantly (*p* > 0.05, *n* = 3).

### 3.4. Effect of Sa-FeNPs on pH of Sediments

**Figure 6:**
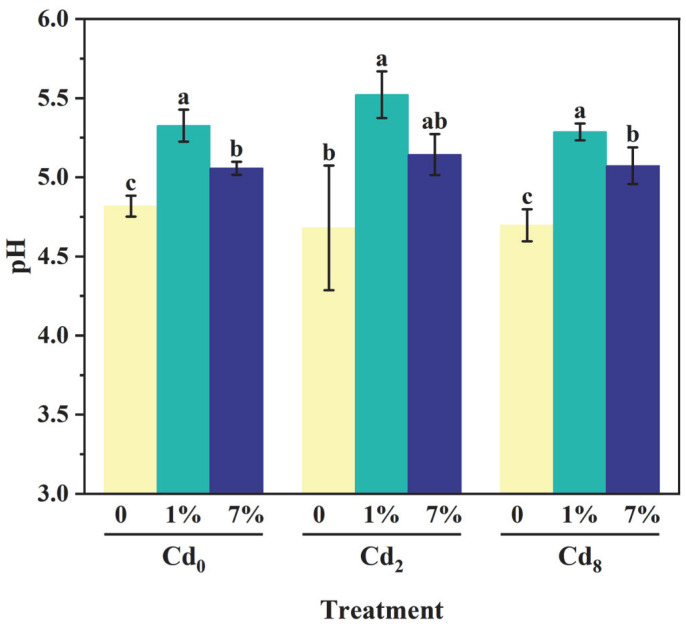
The variations in sediment pH values under different treatments. Different lowercase letters indicate significant differences between different Sa-FeNP treatments under the same Cd treatment as determined by the LSD test (*p* < 0.05, *n* = 3), while the same lowercase letters indicate no significant difference.

The addition of Sa-FeNPs resulted in a significant increase in the pH values of sediments across all three Cd levels (Cd_0_, Cd_2_, and Cd_8_). Under the treatment with 1% Sa-FeNPs, the pH values increased by 0.51, 0.84, and 0.59 for Cd_0_, Cd_2_, and Cd_8_ sediments, respectively (*p* < 0.05, *n* = 3). Under the treatment with 7% Sa-FeNPs, the pH values increased by 0.24, 0.46, and 0.28 for Cd_0_, Cd_2_, and Cd_8_ sediments, respectively (*p* < 0.05, *n* = 3) (Figure 6).

### 3.5. Effect of Sa-FeNPs on Organic Carbon

**Figure 7:**
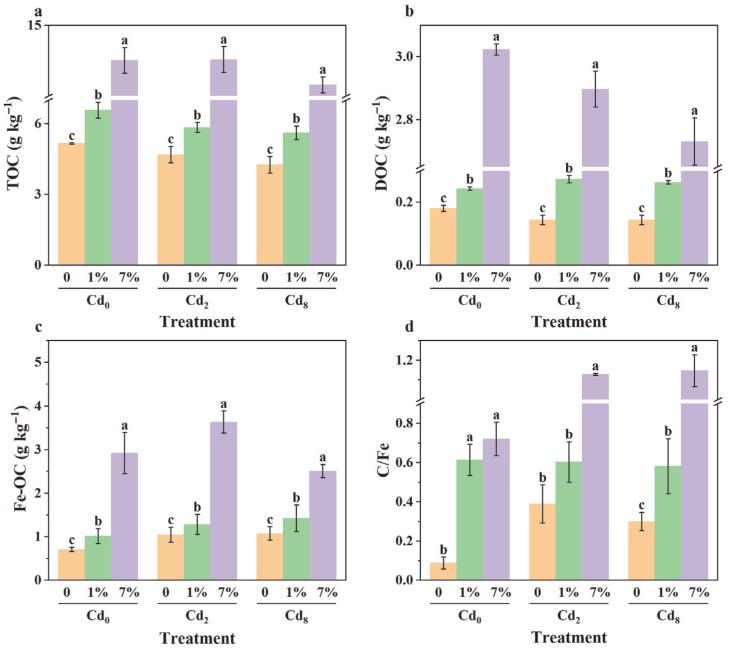
(**a**) The variations in the contents of total organic carbon (TOC), (**b**) dissolved organic carbon (DOC), (**c**) iron-bound organic carbon (Fe-OC), and (**d**) the molar ratio of C/Fe in sediments under different treatments. Different lowercase letters indicate significant differences between different Sa-FeNP treatments under the same Cd treatment as determined by the LSD test (*p* < 0.05, *n* = 3), while the same lowercase letters indicate no significant difference.

As shown in Figure 7a-c, the contents of TOC, DOC, and Fe-OC in the sediments significantly increased with the addition of Sa-FeNPs (*p* < 0.05, *n* = 3). Specifically, under the 7% Sa-FeNP treatment, the TOC, DOC, and Fe-OC contents in Cd_0_ sediment increased by 8.35 mg kg^−1^, 2.84 mg kg^−1^, and 2.21 mg kg^−1^, respectively; in Cd_2_ sediment, they increased by 8.86 mg kg^−1^, 2.75 mg kg^−1^, and 1.75 mg kg^−1^, respectively; and in Cd_8_ sediment, they increased by 8.22 mg kg^−1^, 2.59 mg kg^−1^, and 1.69 mg kg^−1^, respectively. However, the increase in the C/Fe molar ratio of Fe-OC was not statistically significant (*p* > 0.05, *n* = 3).

### 3.6. Correlation Analysis

**Figure 8:**
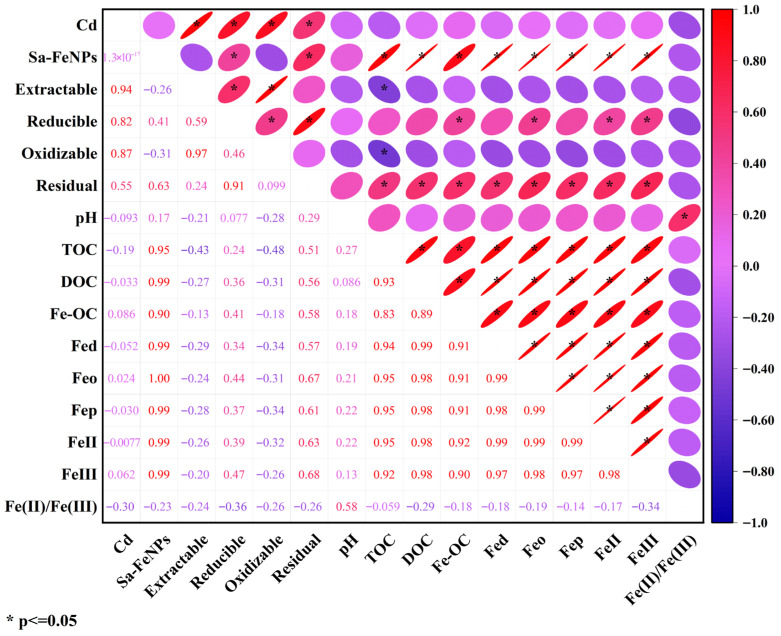
The Pearson correlation coefficients between different Cd fractions and various environmental factors.

The correlations among the variables are presented in Figure 8. The Cd treatment was significantly positively correlated with all four forms of Cd (r = 0.94, r = 0.82, r = 0.87, r = 0.55; *p* < 0.05). Sa-FeNPs exhibited significant positive correlations with reducible Cd (r = 0.41, *p* < 0.05), residual Cd (r = 0.63, *p* < 0.05), TOC (r = 0.95, *p* < 0.05), DOC (r = 0.99, *p* < 0.05), Fe-OC (r = 0.90, *p* < 0.05), Fed (r = 0.99, *p* < 0.05), Feo (r = 1.00, *p* < 0.05), Fep (r = 0.99, *p* < 0.05), Fe(II) (r = 0.99, *p* < 0.05), and Fe(III) (r = 0.99, *p* < 0.05). Weak-acid-extractable Cd and oxidizable Cd were significantly negatively correlated with TOC (r = −0.43, *p* < 0.05; r = −0.48, *p* < 0.05). Reducible Cd showed significant positive correlations with Fe-OC (r = 0.41, *p* < 0.05), Feo (r = 0.44, *p* < 0.05), Fe(II) (r = 0.39, *p* < 0.05), and Fe(III) (r = 0.47, *p* < 0.05). Residual Cd was significantly positively correlated with TOC (r = 0.51, *p* < 0.05), DOC (r = 0.56, *p* < 0.05), Fe-OC (r = 0.58, *p* < 0.05), Fed (r = 0.57, *p* < 0.05), Feo (r = 0.67, *p* < 0.05), Fep (r = 0.61, *p* < 0.05), Fe(II) (r = 0.63, *p* < 0.05), and Fe(III) (r = 0.68, *p* < 0.05). The correlation analysis suggests that the distribution of Cd fractions is closely related to the redox state of Fe and the transformation of TOC.

## 4. Discussion

### 4.1. The Immobilization Efficacy of Sa-FeNPs on Cd

The addition of Sa-FeNPs effectively mitigated Cd’s direct toxicity and bioavailability in the sediments by transforming its speciation, as evidenced by the decrease in weak-acid-extractable Cd and the significant increase in the stable residual fraction (Figure 4). This immobilization efficacy is superior to that of several existing materials. While Sa-FeNPs share the amorphous structure, functional groups, and iron oxide content that typically result from plant-based synthesis, they are distinguished by a greater specific surface area and Fe loading than those derived from tea or eucalyptus [13,27], attributable to the unique antioxidant profile of *S. alterniflora* [17]. In a direct performance comparison, Sa-FeNPs (1% application) reduced the content of weak-acid-extractable Cd by 5.56%, surpassing the 4.30% reduction by citrus peel-based FeNPs [31]. Their smaller particle size (Figure 1) explains this heightened reactivity [32]. Sa-FeNPs boosted the residual Cd fraction by 25.98%, significantly exceeding the 14.94% achieved by crystalline iron oxides [33], owing to their better dispersion and functional group abundance (Figure 2d). The green synthesis protocol operates under ambient conditions, the primary feedstock (*S. alterniflora*) is a free and abundant waste material, and the synthesis process avoids the use of expensive or hazardous chemicals, positioning our method as low-cost and sustainable. In addition, compared with ex situ remediation technology, the in situ application of Sa-FeNPs represents a less destructive and lower-cost treatment strategy for contaminated sediments.

### 4.2. The Mechanism of Sa-FeNPs Immobilizing Cd in Sediments

The correlation analysis (Figure 8) revealed that the stability of Cd is closely linked to Fe and C dynamics, suggesting that Sa-FeNPs immobilize Cd through a synergistic combination of direct retention and the promotion of secondary sequestration pathways. Firstly, direct adsorption and complexation serve as the initial and rapid immobilization mechanisms. The large specific surface area and abundant surface functional groups (e.g., hydroxyl, carboxyl) on the amorphous iron oxides of Sa-FeNPs provide ample sites for Cd^2+^ capture. The surface reactions involved in Cd immobilization are described by Equations (1)–(3) [33,34], showing a process favored by the high affinity between Cd and iron (hydr)oxide surfaces.

Beyond direct surface binding, the transformation and redox cycling of iron play a pivotal role in enhancing long-term Cd sequestration. This is strongly supported by the significant statistical correlations we observed in Figure 8. For instance, the positive correlation between Fe(III) and residual Cd was the strongest (r = 0.68, *p* < 0.05), quantitatively confirming that the iron redox cycle drives Cd to transform into a stable residual form. Sa-FeNPs significantly increased the pools of both Fe(II) and Fe(III) in the sediment (Figure 5d,e); as iron reduction predominantly occurs via microbial activity, we speculate that iron redox cycling is also driven by microbial activity [12]. This process involves the reductive dissolution of amorphous Fe(III) oxides, releasing Fe^2+^ and potentially co-sorbed Cd [35]. Critically, this is not a terminal release but a transformative step. The released Fe^2+^ can reprecipitate as secondary iron minerals [36]. During this recrystallization, Cd ions can be incorporated into the mineral lattice via coprecipitation or isomorphic substitution [37,38], effectively transferring Cd from labile fractions into the highly stable residual pool (Figure 4). Similarly, the strong positive correlation between amorphous iron oxide (Feo) and residual Cd (r = 0.67, *p* < 0.05, Figure 8) highlights the crucial role of these secondary minerals in adsorbing and binding Cd. This transformation explains why the increase in residual Cd far exceeds what could be achieved by adsorption alone.

(1) $$\begin{array}{c} {FeO}^{-}+{Cd}^{2+}\to {FeOCd}^{+} \end{array}$$

(2) $$\begin{array}{c} FeOH+{Cd}^{2+}+H_{2}O\to FeOCdOH+2H^{+} \end{array}$$

(3) $$\begin{array}{c} {Cd\left(OH\right)}^{+}+\equiv Fe-{OH}_{2}^{+}↔\equiv Fe-OCd{\left(OH\right)}^{0}+2H^{+} \end{array}$$

Concurrently, the interplay with organic carbon and pH shifts creates a favorable environment for immobilization. The organic biomolecules (e.g., polysaccharides, phenols) released from Sa-FeNPs increase the total organic carbon (TOC, Figure 7a). The surface of organic carbon is typically negatively charged [39], enabling adsorption reactions with positively charged Cd ions. The microbial decomposition of this organic matter produces dissolved organic carbon (DOC), which can further promote the reductive dissolution of iron oxides, thereby sustaining the regenerative cycle of iron mineral formation [40]. The results of the correlation analysis (Figure 8) further confirmed that DOC is significantly positively correlated with iron and iron oxide. Simultaneously, the hydrolysis of Fe^2+^ (Equations (4) and (5)) and decarboxylation of organic anions (Equation (6)) consume protons, leading to a significant increase in sediment pH (Figure 6) [12,41]. The elevated pH enhances the negative surface charge on both Sa-FeNPs and sediment colloids, strengthening electrostatic attraction to Cd^2+^. Moreover, this favors the formation of amorphous iron oxides, such as iron hydroxides, collectively contributing to more stable and resilient immobilization of Cd.

(4) $$\begin{array}{c} {2Fe}^{2+}+{2H}_{2}O\to {2Fe}^{3+}+H_{2}+{2OH}^{-} \end{array}$$

(5) $$\begin{array}{c} {2Fe}^{2+}+{2H}^{+}+\frac{1}{2}O_{2}\to {2Fe}^{3+}+H_{2}O \end{array}$$

(6) $$\begin{array}{c} R-CO-{COO}^{-}+H^{+}\to R-CHO+{CO}_{2} \end{array}$$

### 4.3. Carbon Sequestration Potential and Future Perspectives

Beyond metal immobilization, our findings suggest that Sa-FeNPs may contribute to sediment carbon stabilization. The substantial quantity of iron oxides generated from Sa-FeNPs can interact with organic carbon via adsorption and coprecipitation to form stable iron-bound organic carbon (Fe-OC, Figure 7c) [42]. In this study, the C/Fe molar ratios in the 1% and 7% Sa-FeNPs treatments were below 1, indicating that the iron oxides primarily stabilized organic carbon through adsorption [43]. This process effectively reduces the bioavailability and decomposition rate of organic carbon, and the dynamic “dissolution-regeneration-refixation” cycle of iron minerals can ensure the persistence of this carbon sequestration effect [44]. This highlights the potential of Sa-FeNPs to serve a dual function, mitigating Cd pollution while simultaneously enhancing the carbon sequestration capacity in coastal wetlands. However, this study lacks direct evidence linking Sa-FeNPs to carbon sequestration (e.g., CO_2_ flux measurements, comparative mineralization rates). Further research is needed to comprehensively evaluate the positive and negative impacts of Sa-FeNPs on the transformation of organic carbon forms and greenhouse gas emissions in sediments.

## 5. Conclusions

Our study confirmed the efficacy of *S. alterniflora*-derived iron nanoparticles (Sa-FeNPs) in immobilizing cadmium in sediments. This immobilization is driven by the transformation of iron oxides and the abundance of functional groups on Sa-FeNPs, which facilitate adsorption and coprecipitation. The concurrent increase in iron-bound organic carbon indicates the potential for achieving coupled pollution remediation and carbon sequestration. Our approach presents an eco-friendly solution to heavy metal contamination while simultaneously proposing a valuable use for an invasive species, thereby transforming a significant ecological burden into a resource for environmental remediation.

## Data Availability

The original contributions presented in this study are included in the article material. Further inquiries can be directed to the corresponding author.

## Abbreviations

The following abbreviations are used in this manuscript:

Fed	Free iron oxides
Feo	Amorphous iron oxides
Fep	Complex iron oxides
TOC	Total organic carbon
OM	Organic matter
DOC	Dissolved organic carbon
Fe-OC	Iron-bound organic carbon
SEM	Scanning electron microscopy
EDS	Energy-dispersive X-ray spectrometry
TEM	Transmission electron microscopy
XRD	X-ray diffraction
XPS	X-ray photoelectron spectroscopy
FTIR	Fourier transform infrared
